# Considerations for Establishing Successful Coronavirus Disease Vaccination Programs in Africa

**DOI:** 10.3201/eid2708.203870

**Published:** 2021-08

**Authors:** Victor Williams, Bassey Edem, Marianne Calnan, Kennedy Otwombe, Charles Okeahalam

**Affiliations:** University of the Witwatersrand School of Public Health, Johannesburg, South Africa (V. Williams, K. Otwombe);; Medical Research Council Unit The Gambia at London School of Hygiene and Tropical Medicine, Fajara, The Gambia (B. Edem);; University Research Co., LLC, Manila, the Philippines (M. Calnan);; University of the Witwatersrand Perinatal HIV Research Unit, Johannesburg (K. Otwombe);; University of the Witwatersrand Graduate School for Business Administration, Johannesburg (C. Okeahalam)

**Keywords:** Africa, coronavirus disease, COVID-19, COVID-19 vaccines, disease control strategies, disease transmission, pandemics, public health logistics, respiratory infections, SARS-CoV-2, severe acute respiratory syndrome coronavirus 2, sub-Saharan Africa, vaccination coverage, vaccination programs, vaccinations, zoonoses, viruses

## Abstract

The accelerated development of coronavirus disease (COVID-19) candidate vaccines is intended to achieve worldwide immunity. Ensuring COVID-19 vaccination is crucial to stemming the pandemic, reclaiming everyday life, and helping restore economies. However, challenges exist to deploying these vaccines, especially in resource-limited sub-Saharan Africa. In this article, we highlight lessons learned from previous efforts to scale up vaccine distribution and offer considerations for policymakers and key stakeholders to use for successful COVID-19 vaccination rollout in Africa. These considerations range from improving weak infrastructure for managing data and identifying adverse events after immunization to considering financing options for overcoming the logistical challenges of vaccination campaigns and generating demand for vaccine uptake. In addition, providing COVID-19 vaccination can be used to promote the adoption of universal healthcare, especially in sub-Saharan Africa countries.

The World Health Organization (WHO) declared the spread of severe acute respiratory syndrome coronavirus 2 (SARS-COV-2), the causative agent of coronavirus disease (COVID-19), a pandemic in March 2020 on the basis of the rapid rate of increase in infections across many countries. As of May 9, 2021, ≈157 million cases and 3.2 million deaths had been recorded globally (*1*), a figure that continues to grow. The COVID-19 pandemic, which originated in Wuhan, China, in late 2019, led to global shutdowns or restrictions of economic and social activities and has caused an unprecedented strain on healthcare services. Although the initial infections were transmitted to local residents from travelers, community infections now make up the bulk of new infections despite ongoing use of preventive measures, including restricted movement, sanitization, face mask wearing, and social distancing based on guidelines or mandates enacted by various governments. New infections continue to occur daily, and many countries anticipate a third wave, which is already underway in a few places. Effectively rolling out programs to distribute available vaccines is urgently needed to complement these ongoing public health measures (*2*,*3*).

When we have limited knowledge of the epidemiology of an infectious disease, waiting to achieve global herd immunity without a vaccine comes at considerable cost because health systems need to increase their use of resources for unplanned expenses to address illness and death. Herd immunity is the indirect protection from an infectious disease acheived in a susceptible population when an adequate proportion of the population becomes immune to the infection (*4*,*5*). Ongoing research has demonstrated that persons who recover from the infection develop antibodies and cellular immune responses that might offer some protection; these findings form the basis for continuing development of COVID-19 vaccines (*6*,*7*). The duration of protection achieved postinfection, although uncertain, is estimated to be ≥6 months (*4*,*6*,*8*).

As of May 7, 2021, WHO had documented 183 candidate vaccines in preclinical evaluation stages and 97 in clinical evaluation, including 22 in phase 3 or 4 trials and ≥8 approved by different national regulatory authorities for emergency or full use (*8**–**10*). Consequently, vaccination of priority groups and the general population has begun on different scales globally including in countries in Africa. Unfortunately, the discovery of multiple COVID-19 variants has raised uncertainty about the efficacy of the available vaccines. Variants have been detected in many countries: United States (B.1.526/B.1.526.1/B.1.427/B.1.429), South Africa (B.1.351), United Kingdom (B.1.1.7/B.1.525), Brazil (P.1/P.2), and India (B.1.617/B.1.617.1/B.1.617.2/B.1.617.3) (*11*).

Considering that successful candidate vaccines will require mass production to meet global demand, governments need to take immediate action to address potential barriers to establishing effective vaccination campaigns in Africa after vaccines are available. Data from WHO and the United Nations Children’s Fund (UNICEF) indicate that, in 2019, Africa had the highest levels of unvaccinated children and the highest number (6.8 million) of children without any doses of the diptheria-pertussis-tetanus1 (DPT1) vaccine (*12*). These data illustrate the challenges facing individual country vaccine programs. However, previous successful vaccination campaigns in Africa provide models from which countries can draw best practices for evidence-informed decision making and planning. MenAfriVac campaigns, introduced in 2010 to provide meningococcal vaccines in the Africa meningitis belt, led to a substantial reduction in meningitis epidemics and a 99% reduction in group A meningococcal meningitis (*13*). The success can be attributed to commitments from multilateral stakeholders to develop and distribute the vaccine and affected countries to roll out vaccination campaigns with efficient surveillance systems simultaneously (*13*). Because the target population for COVID-19 vaccination is broader, country vaccination programs in Africa should consider both universal and unique potential challenges to this effort. Anticipating and understanding these challenges and addressing them through data-driven planning, will be vital to overcome barriers to establishing successful COVID-19 vaccination campaigns that include robust social behavior efforts targeted to residents (Table 1).

**Table 1:** Key considerations for COVID-19 mass vaccination program development*

Category	Key considerations
Vaccine characteristic	Vaccine thermostability and requirement for cold chain system; route of administration; no. doses/person; COVID-19 testing capacity; adverse event surveillance
Financing	Ability of low-and middle-income countries to pay for vaccines and deliver financing
Prioritizing beneficiaries	Priority populations: healthcare workers, first responders, essential services personnel, elderly persons, persons with coexisting conditions, children; use of phased approach with timelines
Vaccination policy and logistics	National policy to guide vaccination process and ensure availability of resources. Timelines for receiving and distributing vaccine from manufacturers after procurement
Communications	Effective communication about benefits of the vaccine to increase acceptability, especially with phased approaches
Vaccine data management systems	Track demographics of vaccinated persons, doses received, risk factors, and adverse events

*COVID-19, coronavirus disease.

## Vaccine Characteristics

Although many COVID-19 vaccines remain in clinical development, ≥8 vaccine candidates have completed phase 3 trials and received emergency authorization: Oxford-AstraZeneca (https://www.astrazeneca.com), Moderna (https://www.modernatx.com), Pfizer-BioNTech (https://www.pfizer.com), Gamaleya (Sputnik) (https://sputnikvaccine.com), Sinovac (CoronaVac) (http://www.sinovac.com), Sinopharm (http://www.sinopharm.com), Novavax (https://www.novavax.com), and Janssen (Johnson & Johnson) (https://www.jnj.com). Moderna and Pfizer report >90% efficacy and Oxford-AstraZeneca 62%–90% when recipients have been fully vaccinated (*9*,*14**–**16*). Candidate vaccines are mainly being developed from mRNA (e.g., Pfizer-BioNTech and Moderna), nonreplicating viral vector (e.g., Oxford-AstraZeneca, Gamaleya, and Janssen), or inactivated virus (e.g., Sinovac and Sinopharm). The logistic requirements vary for distributing different vaccines. Except for the Janssen vaccine, which requires only 1 dose, all of the vaccines require 2 intramuscular doses administered several days apart (*9*). The Pfizer-BioNTech and Moderna vaccines might be challenging to administer in Africa because each requires –70°C cold chain storage and transportation (*14*). 

The WHO target product profile for COVID-19 vaccines specifies that target vaccines should be indicated for active immunization, administered in either a 1- or 2-dose regimen, viable for all age groups in an outbreak, including the elderly, and have a rapid onset of protection, preferably ≤2 weeks (*17*). Also, the vaccine must be prequalified in a multidose vial presentation and be thermostable, which can simplify vaccine distribution and eliminate the additional costs required for cold chain management. Distributing vaccines to target populations in mass campaigns, as postulated, requires a less complex administration route than other distribution strategies. However, mass vaccination campaigns might present some challenges. The elderly constitute an important target population, and inadequate immune response to vaccination in this age group is well documented; in addition, experience with routine immunization of this age group in Africa is limited (*18*). 

Influenza and pneumonia are the vaccines most commonly recommended for elderly persons, and studies on the uptake and effectiveness of these vaccines in Africa are few. Studies from South Africa show that elderly populations at risk for high mortality rates have reduced uptake of the influenza vaccine, indicating the need for more effective approaches to increase uptake and ultimately achieve population-level immunity (*19*). Compared with the influenza vaccine, which showed an efficacy of 30%–50% among persons ≥65 years of age and 70%–90% in children and younger adults, available data from 2 leading vaccine candidates (Pfizer-BionTech and Oxford-AstraZeneca) indicate an efficacy >90% in persons ≥65 years of age (*15*,*16*,*18*). This notable improvement in vaccine design addresses reduced immune response among the elderly. 

## Program Monitoring

One challenge to establishing vaccination programs in Africa is that data from COVID-19 testing has been suboptimal, which might affect rollout of vaccination campaigns. Because of limited testing, information on the number of cases and patient demographics is lacking, further hindering effective planning. Therefore, countries in Africa need to increase testing capacity to better characterize and manage the pandemic. 

Another planning consideration is the capacity to monitor adverse events following immunization (AEFI), which is imperative in the context of a COVID-19 vaccine rollout. First, these vaccines have undergone rapidly accelerated development. The adverse event profiles of the vaccines are not fully known, and early data from trials of 2 leading vaccine candidates suggested more adverse events occurred in the vaccine groups than in the control groups (*15*,*16*). After rollout of the vaccines, adverse events including rheumatoid symptoms, blood clots, severe headache, and fever were reported, but WHO guidance indicates that the benefits of the vaccine outweigh the risks (*20*). Phase 4 studies and robust postmarketing surveillance will strengthen risk profile characterization and help manufacturers and regulators effect risk minimization strategies, vital steps that might have been missed because of the accelerated development and licensing process (*21*).

South Africa is the only country in Africa that has thus far conducted COVID-19 vaccine trials, indicating that safety and efficacy data for the vaccines might be limited in these populations (*14*). AEFI reporting has historically been weak in Africa, using only passive monitoring systems that are restricted to tuberculosis (TB) and HIV. However, AEFI systems are being scaled up, as demonstrated in Malawi and the Democratic Republic of the Congo, where officers are being trained on pharmacovigilance and AEFI with subsequent active follow-up to improve documentation of adverse events from medications and vaccines (*22*,*23*). This process has been recognized by WHO as a best practice and recommended for other countries in Africa. Multistakeholder collaborations are needed to sustain the strengthening of postlicensure AEFI monitoring (*21*) and the national immunization technical advisory groups in each country should identify and address policy issues related to COVID-19 vaccine rollout and administration (*24*).

## Vaccination Logistics

Rollout of a COVID-19 vaccination campaign in Africa will require each country to formulate a national deployment plan, a document that will guide the overall allocation of resources for a successful campaign by ensuring the availability of personal protective equipment, vaccine supply, storage and transportation within a continuous cold chain system (if needed), human resources, security, and other prerequisites (*24*). It can be argued that some of these resources are already available from the WHO Expanded Program on Immunization (EPI) programs in different countries and that COVID-19 vaccination administration programs can easily be integrated. However, existing EPI programs target specific populations, mainly pregnant women and infants, and might not offer adequate resources to accommodate a large-scale population-based vaccination campaign. In addition, existing challenges encountered by EPI programs, such as lack of funding, human resources, logistics infrastructure, transportation to certain difficult-to-access terrains, and kidnappings and killings of EPI program staff in conflict zones in some Africa countries, further compicate the challenges brought by the COVID-19 pandemic (*12*). Therefore, careful assessment of the current state of each country’s EPI will be required to identify critical deficiencies and necessary resources added before existing EPI infrastructures can be integrated into successful COVID-19 vaccination campaigns. Given that larger populations that will need to be vaccinated compared with target groups in previous vaccination campaigns for other diseases, adopting a phased approach based on priorities for vaccinating different population groups in each country could prevent overburdening of existing EPI infrastructures and resources (*13*,*25*).

## COVID-19 Vaccination Program Financing

To prepare for distributing licensed COVID-19 vaccines once they became available, the United States and European Union committed funds for developing programmatic infrastructure and purchasing vaccine doses. Considering the cost of advanced candidate vaccines, uncertainty had been expressed about how developing countries would fund vaccine purchases (*26*). A coalition, COVID-19 Global Access (COVAX), led by the Global Alliance for Vaccines and Immunizations (Gavi), the Coalition for Epidemic Preparedness Innovation, and WHO, was tasked with ensuring equitable distribution of licensed COVID-19 vaccines to give developing countries access to the vaccines through the advance market commitment model (*27*). This model has been used to successfully distribute new vaccines, such as pneumococcal conjugate vaccines, in low- and middle-income countries. COVAX has been proposed to provide COVID-19 vaccine for 20% of the population of each country registered under the WHO fair allocation mechanism (*28*). Since February 2021, >41 Africa countries have received 18 million doses of the COVID-19 vaccine from COVAX to commence vaccination of priority populations; COVAX has committed to distributing additional doses to accommodate more vaccinations. So far, vaccinations in Africa constitute ≈1.5% of vaccinations globally. Observed challenges include low vaccine uptake because of poor administration and vaccine hesitancy, which have resulted in expiration of received vaccines in some countries (*29*,*30*).

Since 2001, the cost of the basic package of vaccines recommended by the United Nations for all children has risen >2,700% (from US $1.38 to $39.00) (*27*) even at the best possible price, paid by the most impoverished countries eligible for support from Gavi. For countries graduating from or not eligible for Gavi assistance, just adding 1 new vaccine to a national EPI program’s mandate would cost US $0.54–$2.34 per person in resources above the vaccine purchase price (*31*). However, variations within and between countries and the uncertain price per dose and wastage rates for the candidate COVID-19 vaccines makes the actual administration costs difficult to predict (*32*). Countries need to develop a financial strategy that, in addition to contributions from COVAX, enables them to procure adequate amounts of the vaccine to ensure access among the beneficiary populations. Considering the number of resources deployed to address the COVID-19 pandemic, reductions in development assistance for health, and economic activity lost because of widespread quarantine, each country needs to carefully identify the resources required for its COVID-19 vaccination program and where to find those resources. Resource requirements will depend on COVID-19 incidence, target vaccination coverage, size of at-risk populations, special demographic and geographic features, availability of existing infrastructure, and competing social priorities (*33*).

In line with the principles of universal health care, vaccination targets should be supported by financial mobilization in a way that ensures resources are available in a timely and reliable manner, do not burden the poor, minimize administrative costs, promote program efficiency, require accountability in resource use, and boost self-sufficiency (*34*). Countries need to consider the pros and cons of each funding source, whether private or public, domestic or external, when making reasoned choices for financing options to achieve vaccination goals of equity, access, use, quality, and safety. For example, depending on out-of-pocket fees to finance immunization services will burden the poor. The type and characteristics of funding mechanisms (Table 2) should also be considered. For example, although trust funds might generate a steady stream of financial resources that can be earmarked for vaccination, they can be administratively costly under certain circumstances. 

**Table 2:** Sources of financing for administering COVID-19 mass vaccination programs*

Type	Domestic	External
Public	Tax revenues (national or subnational) for current spending	Project grants from bilateral or multilateral agencies
	Tax revenues (national or subnational) for repaying domestically or internationally held debt	Grant portion of development loans
		Budget support
	Social health insurance (compulsory)	Debt relief proceeds
		Sectorwide approaches
Private	User fees (out of pocket, direct employer payment)	Vaccine fund
	Cross-subsidies	Project grants from philanthropic institutions
	Health insurance	Contributions (often in-kind) from vaccine manufacturers

*COVID-19, coronavirus disease.

## Prioritizing Vaccine Recipients 

Prioritizing who should receive the COVID-19 vaccine and in what order may create ethical dilemmas. COVID-19 vaccine strategies aim to achieve sufficient coverage to develop population-level immunity. However, the people most susceptible to severe COVID-19 infections are often nearing the end of their economic productivity, have underlying conditions, or both. Although these target populations may be prioritized to receive the vaccine, they often have the weakest immune responses (*18*)*.* However, available data from 2 leading COVID-19 vaccine candidates indicates that, on the contrary, immune responses are similar across all age groups (*15*,*16*). Conversely, groups at high risk for exposure to the virus, such as healthcare providers, supermarket workers, and other frontline workers, are often young and healthy.

Questions persist about whether to prioritize specific population groups hardest hit by the virus. In sub-Saharan Africa, with its youthful populations, high HIV and TB rates, and inadequate resources, decision makers must make ethically justifiable decisions about who receives the COVID-19 vaccine and in what order (*35*) (Table 3). When identifying priority populations, countries should consider the effects of the availability of data in the immunization system on the criteria, such as age, used to identify potential recipients to ensure accurate tracking. Finally, along with the ethical justifications for which populations are prioritized, countries should consider what they need to achieve to maximize the benefit of the vaccine: preventing increased death and illness by vaccinating populations at highest risk for becoming infected and seriously ill or for transmitting the disease to others and maintaining reciprocal obligations with frontline or critical service providers, including those who volunteered to participate in vaccine development trials, to ensure continuity of services and encourage future participation.

**Table 3:** Guiding ethical principles when considering who should receive COVID-19 vaccine (*35*)*

Principle	Description	Practical application
Equality	Equal interest of everyone unless good reasons to justify differential prioritization	May be most appropriate to guide allocation of scarce resources among persons or populations who can be expected to derive the same benefit from the resource (e.g., high-risk populations)
Best outcomes	Allocation according to capacity to do most good or minimize most harm (e.g., saving most possible lives)	May be most appropriate to guide the allocation of scarce resources that confer substantially different benefits to different persons (e.g., groups expected to derive the most benefit)
Prioritize highest risk	Allocation to persons most at risk	May be most appropriate to guide allocation of resources intended to protect those most at risk (e.g., groups most at risk for infection and severe illness)
Prioritize those tasked with helping others	Allocation to persons with certain skills that can save others or because of their participation in helping others (e.g., vaccine trial volunteers)	May be most appropriate to guide allocation of resources to health care workers, first responders, vaccine trial participants, etc.

*COVID-19, coronavirus disease.

## Communicating Accurate Information about COVID-19 Vaccines

Rollout of COVID-19 vaccine programs shows that vaccine hesitancy exists regardless of the proven benefits of vaccination (*36*,*37*). In a 15-country survey on COVID-19 vaccine perceptions in Africa, 60% of respondents believed that the threat posed by COVID-19 is exaggerated, 42% reported that they have been exposed to a lot of disinformation, and 18% (range 4%–38%) would not accept a COVID-19 vaccine (60% of those because they do not trust the safety of the vaccine and 15% because they claim the virus does not exist) (*37*). These concerns and others posed by the emergence of multiple variants of the virus urgently need to be addressed by providing appropriate information to potential vaccine recipients about the benefits of available vaccines, including offering protection against known variants (*38*,*39*).

Scale-up of programs to distribute antiretroviral medication to treat HIV has shown that continuous communication promoting benefits and debunking myths related to a particular treatment are needed to increase acceptance (*40*). Vaccines are preventive and do not have a prolonged course like HIV treatment and therefore urgently need widespread acceptance. Vaccine refusal because of stigma or differing personal, cultural, or religious beliefs has contributed to a resurgence in vaccine-preventable diseases, such as measles, which had been greatly reduced in different regions of the world (*41*,*42*); vaccine refusal poses a threat to COVID-19 vaccine uptake in Africa and other parts of the world. Mass advocacy and information campaigns are needed to address those concerns (*43*,*44*). In response to this need, WHO developed a COVID-19 vaccine communications plan to promote high vaccine acceptance globally. Who is also partnering with global and regional organizations, scientific communities, civil societies, policy- and decision-makers, media, and the general public to achieve this by making regular and easily accessible communication available about potential benefits and risks of the vaccines (*21*,*45*). Countries in Africa should develop country- and culture-specific communication plans in appropriate languages and communication modes to address specific miscommunication and reinforce the use of preventive measures, such as social distancing, handwashing, and face masks, that have been shown to limit transmission of infection. Interim guidance from WHO on risk communication and community engagement readiness and response to COVID-19 provides a step-by-step guide for country teams (*45*).

## Vaccine Data Management Systems

Successful public health interventions require a robust data management system for efficient data collection, analysis, and interpretation to inform planning and admininstration. This necessity was demonstrated during the 2014 Ebola outbreak in West Africa and continues to be used when developing other public health interventions, including those for HIV, TB, and malaria (*46*,*47*). At the onset of the COVID-19 pandemic, different systems were developed by different countries and organizations to document and track the pandemic globally and locally (*1*,*48*). However, challenges arise from duplicate health information systems, systems not being linked to one another or not using unique identifiers for patients, or systems depending on outside donors that are specific to health programs that might not cover all populations in sub-Saharan Africa (*49*). A robust system in which data can be disaggregated into different sociodemographic indices is required for COVID-19 surveillance and tracking related demographics, doses of vaccine administered, adverse events, and underlying conditions in vaccinated persons. Any proposed COVID-19 vaccination data system should be integral to existing country immunization information systems and, where feasible, linked with laboratory information systems to easily verify COVID-19 test results and enhance case-based management (*50*). As COVID-19 vaccines are available, a COVID-19 vaccination certificate might become a requisite for international travel, pending the availability of a cure (*8*). Robust data systems with unique personal identifiers could be used to form a database for identifying and certifying vaccinated persons. Such systems could also support research on vaccine effectiveness. However, the number of doses required for specific vaccines must be considered during planning because of the need to follow up with persons who miss second or third doses. These vaccination information systems will be vital for monitoring, evaluating, and improving surveillance of and response to COVID-19 outbreaks.

## Conclusions

The COVID-19 pandemic, although it has disrupted life and destabilized economies globally, has also presented an opportunity for global leaders to reassess basic healthcare infrastructure and preparedness for and capacity to respond to health emergencies in their countries. In many countries, the COVID-19 pandemic response has revealed weaknesses in leadership and disparities in the health infrastructure available to some residents that might be strengthened through collaboration, cooperation, and communication among all stakeholders.

The scientific community’s ongoing research and efforts to develop vaccines in the shortest possible time is commendable and should receive maximum support from all stakeholders. Equitable access to these vaccines should be guaranteed and distribution and admininstration not hindered by cost or logistic challenges. In addition, each country should identify suitable funding mechanisms to procure the right number of vaccine doses and plan for how to deliver them to its target populations. Countries need to clearly define policies on who will be prioritized for vaccination and develop clear admininstration strategies to support the vaccination campaign. Adequate health information systems for documentation and data management are required to track progress, identify challenges, and provide evidence for admininstrators and policymakers. Communicating with intended beneficiaries of vaccination is of utmost importance and should be done through various media to address concerns and minimize miscommunication. Appropriate emphasis should also be placed on mass education about COVID-19 and vaccination campaign policies. 
